# Protocol for modulating anesthesia delta oscillations using closed loop auditory stimulation

**DOI:** 10.3389/fnhum.2026.1748528

**Published:** 2026-03-09

**Authors:** Clara Pic Roca, Hanieh Bazregarzadeh, Louis Morisson, Antonio Martin, Olivier Verdonck, Steve Gibbs, Jean-Marc Lina, Julie Carrier, Philippe Richebé, Catherine Duclos

**Affiliations:** 1 Center for Advanced Research in Sleep Medicine, Centre Intégré Universitaire de Santé et des Services Sociaux du Nord-de-l’Île-de-Montréal, Montreal, QC, Canada; 2 Department of Neuroscience, Faculty of Medicine, Université de Montréal, Montreal, QC, Canada; 3 Hôpital Maisonneuve-Rosemont, Centre Intégré Universitaire de Santé et des Services Sociaux de l’Est-de-l’Île-de-Montréal, Montreal, QC, Canada; 4 Department of Anesthesiology and Pain Medicine, Université de Montréal, Montreal, QC, Canada; 5 Department of Electrical Engineering, École de Technologie Supérieure, Montreal, QC, Canada; 6 Departement of Psychology, Faculty of Arts and Sciences, Université de Montréal, Montreal, QC, Canada; 7 Department of Anesthesiology, Polyclinique Bordeaux Nord Aquitaine, Bordeaux, France

**Keywords:** Bispectral Index, closed-loop auditory stimulation, delta oscillations, general anesthesia, high-density EEG, nociception level index, propofol

## Abstract

**Introduction:**

Delta waves (0.1–4 Hz) are a hallmark of unconsciousness in both sleep and general anesthesia (GA). Closed-loop auditory stimulation (CLAS) delivers brief sounds phase-locked to ongoing slow oscillations and can modulate slow-wave activity (SWA) during sleep, but its efficacy and translational relevance under propofol anesthesia, particularly in the presence of nociceptive input, remain unknown.

**Methods:**

We describe a prospective, within-subject protocol in 30 neurologically healthy adults undergoing elective surgery under propofol GA. Intraoperative high-density electroencephalography (hd-EEG; 128 channels) is recorded while a real-time CLAS algorithm detects *δ*-waves at the Fz electrode, and triggers pink-noise bursts in three conditions: *in-phase* (peak-locked), *anti-phase* (trough-locked), and *sham*. An extended *in-phase* block is then combined with a standardized tetanic nociceptive stimulus (100 Hz, 70 mA, 30 s) to test robustness under an arousal challenge. Depth of hypnosis is monitored with the Bispectral Index (BIS), and nociceptive/autonomic reactivity with the Nociception Level (NOL) index. Primary outcomes quantify *δ*-wave morphology and SWA (amplitude, slopes, duration/frequency, transition frequency, density, power spectral density), along with topography, source estimation, and propagation. Secondary outcomes include depth of anesthesia (BIS), nociceptive balance (NOL), functional connectivity (weighted and directed phase-lag indices), graph-theoretical organization (global efficiency, clustering, modularity, small-worldness), and EEG microstates (duration, coverage, transitions).

**Anticipated results:**

We hypothesize that *in-phase* CLAS will increase amplitude, slope, duration, and density of *δ*-waves, and decrease frequency and transition frequency relative to *sham*, whereas *anti-phase* CLAS will cause a disruption these parameters relative to *sham*. Under nociception, *in-phase* CLAS is expected to these parameters preserve *δ*-wave integrity, with effects attenuated relative to non-nociceptive conditions. We anticipate BIS values to remain within the surgical range but trend lower during *in-phase* CLAS, alongside reduced nociceptive/autonomic responses. At the network level, we expect CLAS-induced *δ*-wave reinforcement to be associated with more locally coherent δ-band activity and reduced frontoparietal integration, consistent with deeper unconsciousness.

**Discussion:**

If confirmed, these findings would position CLAS as a candidate neuromodulation strategy to reinforce anesthetic *δ*-wave dynamics and help stabilize anesthesia without necessarily increasing pharmacological dose, while also providing a systems-level test of the role of slow oscillations in sustaining unconsciousness.

## 1 Introduction

### 1.1 From sleep to anesthesia: a shared neurophysiological landscape

General anesthesia (GA) is often colloquially described as “falling asleep.” Although sleep and anesthesia arise from distinct mechanisms, they share striking neurophysiological signatures. Both are associated with a reversible loss of awareness, diminished sensory processing, and unresponsiveness to external stimuli (Brown et al., 2010; Murphy et al., 2011; Purdon et al., 2013). A central commonality is the presence of synchronized low-frequency activity in the electroencephalogram (EEG), most prominently within the delta frequency range (0.1–4 Hz) (Massimini et al., 2004; Steriade et al., 1993). This convergence has motivated extensive comparisons between anesthesia, sleep, and coma in the neuroscience of consciousness (Akeju and Brown, 2017; Bonhomme et al., 2016; Boveroux et al., 2010; Mashour, 2024; Mashour and Hudetz, 2018).

Within the delta band, two related but distinct phenomena can be identified: slow oscillations (SOs; <1 Hz) and slow waves (SWs; 1–4 Hz) (Massimini et al., 2004; Steriade et al., 1993). SOs are large-amplitude, <1 Hz oscillations representing the alternation between cortical up- and down-states, and are considered the fundamental rhythm of deep non-rapid eye movement (NREM) sleep (Sanchez-Vives and McCormick, 2000; Steriade et al., 1993a, 1993b). In contrast, SWs are faster oscillations in the delta band that often co-occur with SOs but may involve partially distinct synchronization mechanisms, especially thalamocortical contributions (Timofeev and Chauvette, 2011). Together, SOs and SWs form the backbone of the EEG slow-wave activity (SWA) that typifies both deep sleep and anesthesia. For clarity, in the remainder of this manuscript we will use the term “*δ*-waves” to collectively refer to SOs and SWs, unless explicitly distinguished. Here we adopt a slightly broadened delta range (0.1–4 Hz) to encompass very slow fluctuations approaching the classical SO band.

### 1.2 SOs and SWs in anesthesia

Loss of behavioral responsiveness during anesthesia is often accompanied by enhanced SO and SW activity, particularly under the GABAergic agent propofol (Purdon et al., 2013; Murphy et al., 2011). As established in sleep research, SOs and SWs represent distinct but interacting components of delta-band activity, relying on partially different synchronization mechanisms. Under anesthesia, these same oscillatory motifs are preserved in form but are generated and stabilized through pharmacological rather than endogenous network dynamics (Brown et al., 2011; Franks, 2008; Murphy et al., 2011).

In natural sleep, SOs are generated primarily within the neocortex through recurrent excitatory–inhibitory interactions that produce alternating up-states of widespread depolarization and down-states of near-silence across large neuronal populations (Sanchez-Vives and McCormick, 2000; Steriade et al., 1993b). These oscillations propagate as traveling waves across the cortical surface, typically in an anterior-to-posterior direction (Cox et al., 2014; Massimini et al., 2004). While the thalamus is not required for SO initiation, it contributes to their synchronization and amplification via thalamocortical loops (Timofeev and Chauvette, 2011). Under propofol anesthesia, SOs continue to reflect large-scale cortical up–down state alternations, but their dynamics are pharmacologically enforced rather than self-organized. Propofol potentiates GABA-A–mediated inhibition, stabilizing cortical bistability and prolonging down-states, resulting in highly regular, high-amplitude SOs (Brown et al., 2011; Franks, 2008). These anesthetic SOs display striking similarities to those observed during deep NREM sleep, including frontal predominance and anterior-to-posterior propagation, yet with more diffuse initiation sites and stronger interhemispheric symmetry (Franks and Zecharia, 2011; Massimini et al., 2004; Murphy et al., 2011; Lee et al., 2013).

In contrast, SWs rely more often on thalamocortical mechanisms. In sleep, SWs are frequently nested within an SO cycle but reflect higher-frequency delta activity shaped by thalamocortical bursting: hyperpolarization of thalamic relay neurons promotes rhythmic low-threshold calcium spikes that reinforce cortical rhythms (Steriade et al., 1993b; Timofeev and Chauvette, 2011). Under GABA-A-potentiating agents such as propofol, this thalamocortical drive is further stabilized by drug-induced hyperpolarization (Franks, 2008; Brown et al., 2011). Some propofol studies have reported that SWs may originate from more spatially diffuse cortical hotspots and to exhibit more regular temporal dynamics compared with natural NREM sleep (Murphy et al., 2009; Murphy et al., 2011).

### 1.3 General anesthesia: pharmacology and monitoring

GA is a pharmacologically induced, reversible state that enables surgery by combining four key components: hypnosis (loss of consciousness), analgesia (absence of pain), amnesia (lack of memory), and immobility (muscle relaxation) (Brown et al., 2010; Sessler et al., 2012). The concept of balanced anesthesia is to achieve these components through the synchronized administration of smaller, safer doses of multiple agents, thereby reducing the adverse effects that arise from high doses of a single drug (Lundy, 1926; Sessler et al., 2012).

Among these agents, propofol is the most widely used intravenous anesthetic for induction and maintenance of hypnosis. Its primary mechanism is potentiation of the inhibitory GABA-A receptor, leading to neuronal hyperpolarization and reduced excitatory transmission across cortical and thalamic networks (Brown et al., 2010; Franks, 2008). Propofol reliably produces unconsciousness and is strongly associated with the enhancement of SWA, though its effects are dose-dependent: higher concentrations prolong down-states, increase the risk of burst suppression, and can cause cardiovascular depression (Murphy et al., 2011; Purdon et al., 2013).

During GA, particularly its hypnotic and amnestic components, there is no direct clinical sign that reliably indicates the depth of anesthesia. In routine practice, anesthesiologists therefore rely on measured concentrations of volatile anesthetic agents or on predicted effect-site concentrations for intravenous drugs to titrate anesthetic depth. While these pharmacokinetic parameters provide a rough estimate of drug delivery and effect, they do not directly reflect the underlying neural state of unconsciousness, which can vary considerably between individuals despite similar concentrations. To complement these measures, several EEG-based neuromonitoring devices have been developed to provide real-time indices of anesthetic depth.

The Bispectral Index (BIS; Rampil, 1998; Sigl and Chamoun, 1994) is one of the most widely used EEG-based monitors. The BIS algorithm extracts spectral and bispectral features from a frontal EEG signal, quantifying both the power distribution and phase relationships between frequency components, and integrates these into a single index ranging from 0 (isoelectric EEG) to 100 (fully awake). Clinically, values between 40 and 60 are typically targeted to maintain surgical anesthesia. While BIS monitoring has proven useful in reducing the risk of awareness and optimizing anesthetic titration, it remains an indirect measure of brain state, reflecting correlates of anesthetic depth rather than the underlying neuronal mechanisms of unconsciousness. The SedLine^®^ (Masimo Corporation, Irvine, CA, United States) system provides an alternative EEG-based approach to monitoring anesthetic depth. It uses a four-channel frontal EEG montage to compute the Patient State Index (PSI), a 0–100 scale with lower values indicating deeper hypnosis (Drover et al., 2002; Drover and Ortega, 2006; Prichep et al., 2004).

To maintain safe and effective anesthesia, and intraoperative nociception, clinicians traditionally monitor various physiological and clinical markers such as changes in heart rate and blood pressure, alterations in respiratory rate and pattern, pupil size and reactivity, the presence of lacrimation or sweating, variations in muscle tone or spontaneous movement, and other autonomic responses such as flushing or tachycardia. In parallel, some clinicians have started using an emerging device called the Nociception Level (NOL) index, a finger sensor which integrates autonomic signals, including heart rate variability, skin conductance, and photoplethysmography, to provide a real-time estimate of nociceptive processing during surgery (Ben-Israel et al., 2013).

Together, EEG-based indices of hypnotic depth (BIS and PSI) and autonomic measures of nociception (NOL) allow anesthesiologists to titrate anesthetic and analgesic agents in a patient-specific manner, maintaining a safe balance between unconsciousness and analgesia while minimizing complications such as hemodynamic instability, intraoperative awareness, or delayed recovery (Garcia-Larrea and Bastuji, 2018; Mashour et al., 2012; Willingham et al., 2014).

### 1.4 Interactions between nociception and consciousness

In GA, each component interacts dynamically together rather than acting independently. In particular, nociception and consciousness are biologically intertwined. While unconsciousness prevents the subjective experience of pain, nociceptive input continues to activate ascending neural pathways, influencing cortical and subcortical dynamics even under anesthesia (Garcia-Larrea and Bastuji, 2018).

The primary pathway for nociceptive processing originates with peripheral nociceptors, which transmit signals through the spinothalamic tract to the thalamus and subsequently to cortical regions including the insula, the anterior cingulate cortex, and somatosensory cortices (Apkarian et al., 2005). Importantly, these same structures contribute to arousal regulation and awareness, meaning that nociceptive signaling can act as an arousal stimulus capable of modulating the depth of hypnosis (Brown et al., 2010; Garcia-Larrea and Bastuji, 2018). Functional imaging and EEG studies have shown that nociceptive input during anesthesia can transiently increase cortical responsiveness and disrupt ongoing SW dynamics, suggesting a mechanistic basis for nociception-induced lightening of unconsciousness (Bonhomme et al., 2016; Plourde et al., 2006).

This interaction has direct clinical consequences. Inadequately controlled nociception during anesthesia can provoke sympathetic activation, leading to hemodynamic disturbances such as hypertension and tachycardia (Brown et al., 2010). Conversely, excessively high doses of hypnotics used to suppress nociceptive arousals can result in cardiovascular depression and burst suppression, both of which are associated with poor postoperative outcomes (Sessler et al., 2012; Willingham et al., 2014). Thus, modern balanced anesthesia requires continuous assessment of both hypnotic depth and nociceptive state, underscoring the clinical importance of integrating monitors such as the BIS and the NOL index (Ben-Israel et al., 2013; Lewis et al., 2019).

### 1.5 Closed-loop auditory stimulation as a modulator of delta waves

SWA plays critical roles in sleep physiology, including memory consolidation, metabolic clearance, and the dissipation of homeostatic sleep pressure (Fultz et al., 2019; Krueger et al., 2016). Declines in SWA are consistently linked to aging and neuropsychiatric disorders such as depression, schizophrenia, and dementia (Baglioni et al., 2016; Scullin, 2013). Over the past decade, closed-loop auditory stimulation (CLAS) has emerged as a powerful method to modulate SWA in real time. By delivering auditory stimuli phase-locked to ongoing SOs, CLAS can selectively enhance their amplitude and slope when applied on the ascending phase of SOs, right before the positive peak (called “*in-phase*” or “*peak*” stimulation; Ngo et al., 2013; Ngo et al., 2015). Conversely, stimulation during the descending phase, after the positive peak but before the negative peak (called “*anti-phase*” or “*trough stimulation*”) tends to disrupt SWA expression (Navarrete et al., 2020; Ngo et al., 2013). Thus, the precise timing of auditory stimulation is crucial: it can either reinforce or attenuate the manifestation of SOs, depending on the phase of delivery. Importantly, CLAS effects have been shown to be primarily stimulus-locked and self-limited: phase-locked auditory clicks transiently amplify the targeted SO, but do not induce sustained modulation beyond the directly stimulated oscillatory cycles (Ngo et al., 2013; Ngo et al., 2015).

Across sleep studies, *in-phase* CLAS has been shown to modulate slow oscillatory activity and, in several cases, enhance sleep quality and declarative memory consolidation (Diep et al., 2021; Ngo et al., 2013; Ngo et al., 2015; Ong et al., 2016; Papalambros et al., 2017). However, other studies have reported no significant effects on memory outcomes (Henin et al., 2019; Ong et al., 2018), suggesting that the cognitive benefits of CLAS may depend on task type, stimulation parameters, and individual responsiveness. In a multi-night paradigm, repeated CLAS was associated with improvements in sleep continuity and memory performance, and with stimulation-response–dependent associations with plasma amyloid biomarkers (Aβ42/Aβ40 ratio), suggesting that sustained SW reinforcement can engage sleep-dependent physiological processes beyond acute EEG modulation (Zeller et al., 2024).

The effectiveness of this technique relies on the intact transmission of auditory signals through the peripheral and central auditory pathways, beginning with cochlear activation and propagation via the vestibulocochlear nerve (cranial nerve VIII) to higher-order cortical regions (Bellesi et al., 2014; Esfahani et al., 2023; Ngo et al., 2013). Studies suggest that these signals ascend predominantly through the non-lemniscal pathway and reach higher-order associative regions, where they evoke event-related potentials in response to sound (Atienza et al., 2001; Hu, 2003; Jourde et al., 2023). Previous EEG and fMRI studies have confirmed that the brain retains its ability to process auditory stimuli during deep states of anesthesia (Hartikainen and Rorarius, 1999; Nourski et al., 2017; Plourde et al., 2006). These findings suggest that the necessary sensory and cortical pathways required for CLAS remain functional under anesthesia, providing a neurophysiological basis for exploring whether CLAS can effectively modulate anesthetized brain states.

Auditory stimulation is particularly well suited for closed-loop modulation of SOs under anesthesia for several reasons: auditory stimulation at low intensity offers a simple, non-invasive, and temporally precise approach for engaging slow oscillatory dynamics, with minimal interference with the surgical field. In sleep, auditory stimuli are well known to evoke K-complex–like responses that share core morphological and generative features with SOs and rely on corticothalamic circuitry involved in *δ*-wave generation (Cash et al., 2009; Colrain, 2005). Under propofol anesthesia, early auditory cortical responses and thalamocortical relay activity are suppressed but not abolished, providing a preserved pathway through which phase-locked auditory input may interact with drug-stabilized δ oscillations. This preserved auditory–corticothalamic coupling provides a mechanistic rationale for using auditory stimulation, rather than other sensory modalities, to probe and modulate slow oscillatory activity under general anesthesia (Ngo et al., 2013; Nourski et al., 2017).

The mechanistic differences between SWs and SOs are directly relevant for interpreting CLAS effects under anesthesia. Whereas SOs primarily index large-scale cortical synchronization and traveling-wave dynamics, SWs reflect the strength and stability of thalamocortical bursting and cortico-thalamic coupling. Consequently, CLAS-induced changes in δ-wave expression may differentially reflect modulation of cortical up–down state timing, thalamocortical engagement, or their interaction, depending on which oscillatory features are most affected.

In the present protocol, SOs and SWs are not separated, and CLAS targets *δ*-waves broadly within the 0.1–4 Hz range. This choice reflects both methodological and physiological considerations. To date, CLAS has not been applied under general anesthesia, and no prior work has established whether SOs and SWs can or should be differentially targeted in this pharmacologically stabilized state. Accordingly, the present protocol targets *δ*-waves to capture the full spectrum of propofol-induced slow activity and maximize sensitivity to stimulation effects in this unexplored context. Nevertheless, distinguishing between these oscillatory components remains conceptually important for interpreting whether CLAS preferentially modulates cortical synchronization, thalamocortical drive, or a combined contribution to general anesthesia unconsciousness.

### 1.6 Translating CLAS beyond natural sleep

Recent work has extended CLAS beyond natural sleep into pharmacological sedation, demonstrating its potential to modulate *δ*-waves in altered states of consciousness. The CLASS-D protocol (Guay et al., 2021) is a within-subject, crossover, controlled trial in healthy volunteers designed to test whether SWA under dexmedetomidine sedation can be enhanced via closed-loop acoustic stimulation (*in-phase, anti-phase*, and *sham* conditions), with high-density EEG recording and assessments of subsequent sleep architecture. The more recent study by Smith et al. (2024) implemented that protocol and reported that daytime dexmedetomidine sedation paired with *in-phase* CLAS alters slow-wave homeostasis: specifically, it induced sleep-like states that fulfilled certain homeostatic NREM sleep needs, reduced N3 duration on the post-sedation night, and modified dissipation of slow-wave pressure compared to *anti-phase* CLAS. Thus, while Guay et al. (2021) laid out the methods and aims, Smith et al. (2024) provide empirical evidence that those methods produce measurable effects. Together, these studies indicate that CLAS can not only be feasibly applied under sedation with dexmedetomidine but also meaningfully alter SW dynamics and sleep homeostasis. Unlike sedation, GA with propofol represents a deeper state of unconsciousness with stronger drug stabilization and routine nociceptive input, making it a critical testbed for evaluating whether CLAS can reliably enhance *δ*-waves under surgical conditions.

### 1.7 Study rationale

CLAS can augment SWs in sleep (Malkani and Zee, 2020; Ngo et al., 2013; Zeller et al., 2024) and has shown feasibility during pharmacological sedation using dexmedetomidine (Guay et al., 2021; Smith et al., 2024), but it remains unknown whether CLAS can enhance SWA under propofol GA, which is a deeper, drug-stabilized state that routinely includes nociception.

Mechanistically, CLAS is hypothesized to interact with propofol-induced *δ*-waves by moving through preserved auditory-corticothalamic pathways to deliver precisely timed stimulations to ongoing cortical activity. Auditory stimuli evoke time-locked cortical responses that transiently engage corticothalamic circuitry implicated in *δ*-wave generation. When delivered during the ascending phase of *δ*-waves, this stimulation is expected to bias the timing and synchronization of cortical up-down state transitions, increasing δ-wave amplitude. Conversely, stimulation delivered during the descending phase is expected to interfere with these transitions, leading to attenuation of the δ-wave amplitude. Under propofol anesthesia, where cortical and thalamocortical networks are pharmacologically stabilized, such phase-specific perturbations provide a principled mechanism by which CLAS may modulate δ-wave dynamics without increasing anesthetic dose.

Demonstrating SWA enhancement with CLAS during propofol anesthesia, without increasing hypnotic dose and while preserving physiological stability (hemodynamics, oxygenation), would suggest a viable non-pharmacological method to support balanced anesthesia.

### 1.8 Objectives and hypothesis

The primary objective of this study is to determine whether CLAS can modulate *δ*-waves (0.1–4 Hz) during propofol-induced GA. Our central hypothesis is that CLAS can effectively enhance δ-waves during surgical anesthesia, and that this modulation will co-occur with changes in EEG/network features commonly observed in more stable anesthetized dynamics, while remaining inferential with respect to consciousness. While propofol enforces *δ*-waves dynamics via GABA-A potentiation (Brown et al., 2011; Franks, 2008), nociceptive input can still engage ascending pathways and modulate cortical state, challenging the maintenance of hypnosis (Brown et al., 2010; Garcia-Larrea and Bastuji, 2018). Consequently, our secondary objective is to assess whether the modulatory effects of *in-phase* CLAS on *δ*-waves during general propofol anesthesia are preserved in the presence of nociceptive inputs. Because nociception has been shown to interact with cortical arousal systems and transiently disrupt slow-wave dynamics (Bonhomme et al., 2016; Garcia-Larrea and Bastuji, 2018; Plourde et al., 2006), demonstrating that CLAS maintains or augments SWA under these conditions would provide critical translational evidence for its potential intraoperative application.

We will test these objectives through the following specific aims:

*Specific Aim 1 (SA1): Characterize the effect of in-phase and anti-phase CLAS on δ-wave morphology and topography, relative to sham stimulation*. We hypothesize that *in-phase* CLAS will increase amplitude, slope, duration, and density of δ-waves, decrease frequency and transition frequency, and will increase SWA power compared to *sham*. This effect will be most pronounced over frontal derivations, and will propagate posteriorly. We hypothesize that *anti-phase* CLAS will disrupt the intrinsic timing and propagation of δ-waves compared with *sham*, resulting in reduced amplitude, flatter slopes, lower transition frequency and decreased SWA power.

*Specific Aim 2 (SA2): Characterize the effect of in-phase CLAS on depth of anesthesia, as assessed through clinical monitoring tools and high-density EEG*. We hypothesize that *in-phase* CLAS will be associated with EEG and BIS changes indicative of altered anesthetic depth. At the network level, we expect *in-phase* CLAS to be associated with decreased functional connectivity within fronto-parietal circuits, reduced network integration, and increased modular segregation, consistent with a transition toward deeper unconsciousness. Microstate analyses are expected to reveal longer mean durations and reduced repertoire diversity, further reflecting a state of reduced dynamical complexity.

*Specific Aim 3 (SA3): Characterize the effect of in-phase CLAS on δ-waves, in the presence of painful stimulation*. We hypothesize the *in-phase* CLAS will enhance SWA in the presence of nociceptive stimulation, mitigating nociception-induced reductions in *δ*-wave dynamics. We expect this enhancement to be less pronounced than under non-nociceptive conditions, yet sufficient to mitigate nociception-induced reductions in δ-wave dynamics. Specifically, we will assess δ-wave amplitude, slope, frequency, transition frequency, duration, and density, as well as the preservation of spatial coherence across fronto-central regions. Such findings would suggest that CLAS can stabilize cortical δ-waves and counteract arousal-related desynchronization, thereby supporting anesthetic stability and analgesic protection.

## 2 Materials and equipment

### 2.1 Methods

#### 2.1.1 Ethics and study design

This prospective study has been reviewed and approved by the Research Ethics Board of the Centre Intégré Universitaire de Santé et des Services Sociaux du Nord-de-l’Île-de-Montréal (CIUSSS-NIM; MP-12-2022-2984) and Centre Intégré Universitaire de Santé et des Services Sociaux de l’Est-de-l’Île-de-Montréal (CIUSSS-EST; MEO-12-2023-2472). Data will be collected at the Hôpital Maisonneuve-Rosemont (CIUSSS-EST, Montreal, Canada) and Hôpital du Sacré-Cœur-de-Montréal (CIUSSS-NIM, Montreal, Canada).

#### 2.1.2 Participants

Using a within-subject study design, this study will enroll 30 neurologically healthy patients scheduled for elective surgery under GA. The sample size is justified based on feasibility, prior literature, and the within-subject design of the study. Patients will be included if they are aged 18–80 years, comprehend French or English, have an American Society of Anesthesiology (ASA) score of I to III, and are scheduled for major elective surgery (abdominal, gynecologic, urologic, thoracic, or orthopedic) requiring more than 60 min of GA. Patients will be excluded for unstable coronary pathology, heart rhythm disorder, emergency or brain surgery, pregnancy, preoperative hemodynamic failure, substance use disorder (alcohol, drugs, opioids as defined by the DSM-V) in the last 3 months, daily use of opioids in the last 7 days, chronic pain conditions, severe psychiatric pathologies, or epilepsy.

Patients will be contacted by phone within 7 days prior to their surgery by a member of the study team who is not a member of the patient’s treating team. Details of the study will be explained to them, and all questions answered by a member of the study team, who will also verify inclusion/exclusion criteria. The information and consent form, as well as an illustrative document explaining study procedures, will be sent to eligible patients by email. The morning of the surgery, a member of the research team will meet the patients to address any concerns and written informed consent will be obtained.

#### 2.1.3 CLAS algorithm development

Our team has designed a CLAS algorithm for the real-time detection of *δ*-waves, which is directly integrated into an hd-EEG system (128-channel MagstimEGI), using custom Python scripts (Python 3.12.0). Online detection uses a single frontal channel (Fz) sampled at 500 Hz and filtered with a low-pass FIR filter plus moving average to achieve low-latency triggering (20 ms delay). Fz was selected to replicate established CLAS paradigms in sleep (Diep et al., 2021; Dudysová et al., 2024; Navarrete et al., 2020; Ngo et al., 2013; Ong et al., 2016; Papalambros et al., 2017) and because slow oscillations under propofol anesthesia exhibit frontal predominance and preferential involvement of medial cortical regions, with origins and propagation patterns aligned along mesial components of the default mode network (Murphy et al., 2011). For algorithm development and real-time visualization, the CLAS software simultaneously logs 20 additional channels (1–20). Simultaneously, NetStation software (MagstimEGI) acquires the full 128-channel raw EEG for offline analyses.

The CLAS software will deliver one of three types of stimulation: *in-phase*, in the up-slope or up-state of the δ-waves, aiming to be phase-locked with the peak of the δ-waves, *anti-phase*, in the down-slope or down-state of the δ-waves, aiming to be phase-locked with the trough of the δ-waves, and *sham*, where no stimulation happens, as a baseline.

#### 2.1.4 δ-wave detection

All δ-waves are defined according to modified version of standard sleep slow waves criteria (Bouchard et al., 2021).

**Figure 1:**
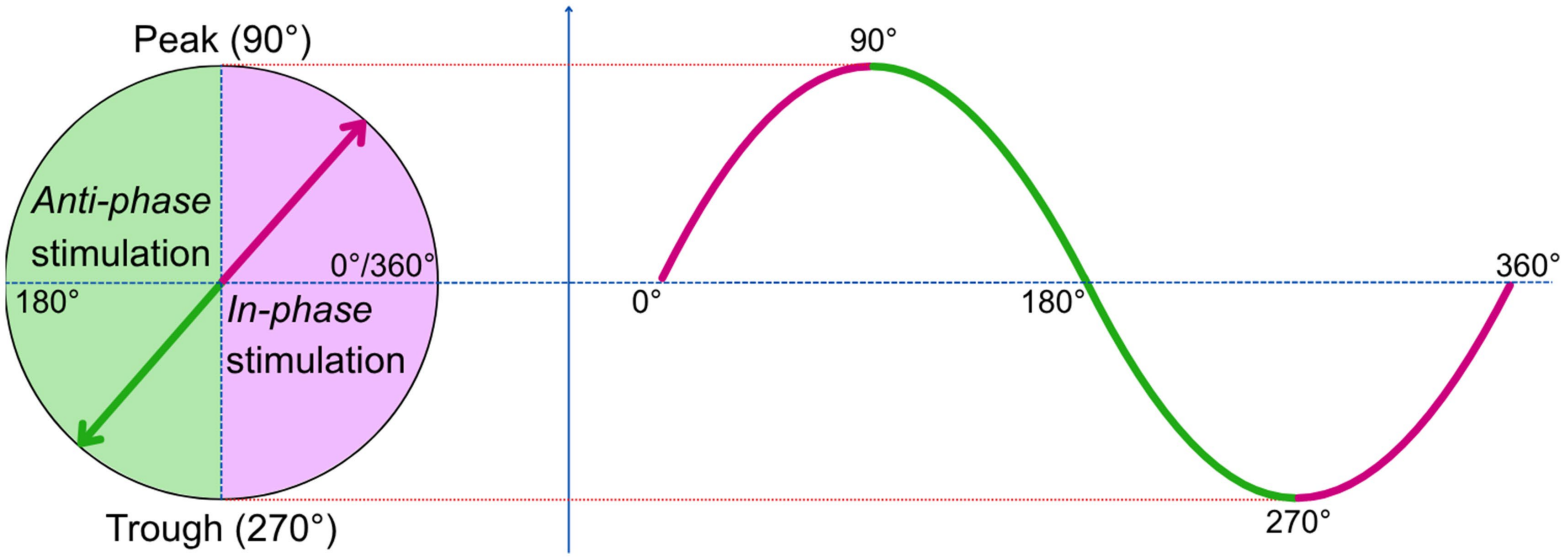
Delta-wave degrees. Schematic sinusoidal wave illustrating the phase-based triggering of CLAS. In-phase stimulation is delivered during the up-slope (270–90°), whereas trough stimulation is delivered during the down-slope (90–270°). These directional, phase-specific effects allow CLAS to either enhance (*in-phase*) or disrupt (*anti-phase*) δ-wave expression.

*In-phase*: δ-wave peaks are detected when the following conditions are met: (1) the signal goes below −40 μV; (2) the signal then crosses 0 μV; (3) the difference in amplitude between the minimum (trough) and the current oscillatory activity is greater than 75 μV; and (4) the peak occurs between 500 and 2,000 ms after the detected trough. The auditory stimulation is triggered during the up-slope of the wave, between 270 and 90° (Figure 1). After an auditory stimulation has been triggered, *in-phase* stimulation is deactivated until the filtered signal passes 0 μV again.

*Anti-phase*: δ-wave troughs are detected when the following conditions are met: (1) the signal goes above 40 μV; (2) the signal then crosses 0 μV; (3) the difference in amplitude between the maximum (peak) and the current oscillatory activity is greater than 75 μV; and (4) the trough occurs between 500 and 2,000 ms after the detected peak. The auditory stimulation is triggered during the down-slope of the wave, between 90 and 270° (Figure 1). To avoid multiple detection within the same slow oscillation, the detection is paused until the filtered signal passes through 0 μV.

#### 2.1.5 Auditory stimuli

The auditory stimuli are composed of binaural bursts of pink noise, also known as 1/f noise. The auditory stimuli last for 50 ms, with a 5 ms rising and falling slope respectively, and are delivered through a trigger box connected to the CLAS detection algorithm. Sound volume will be set at a maximum of 65 dB SPL at the ear canal (Henin et al., 2019).

#### 2.1.6 Pre-anesthesia procedure

**Figure 2:**
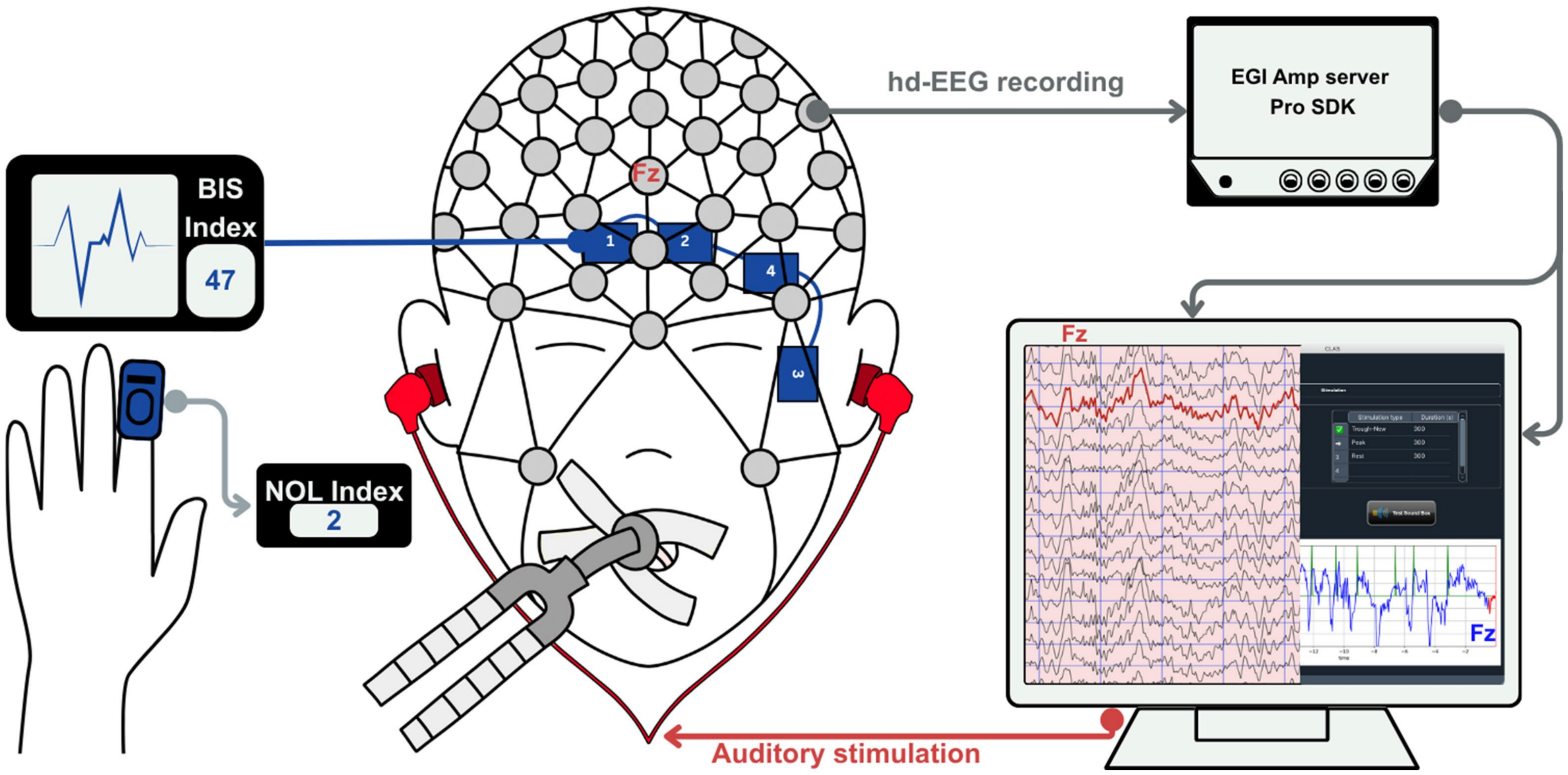
Simplified closed-loop auditory stimulation (CLAS) setup. A high-density EEG cap (128 channels) records neural activity, while a single frontal channel (Fz) is used online by the CLAS algorithm for real-time δ-wave detection and stimulation triggering. The Bispectral Index (BIS^®^) provides continuous monitoring of hypnotic depth, and the Nociception Level (NOL^®^) index monitors nociceptive balance. The full EEG is simultaneously recorded in NetStation (MagstimEGI, v5.4.3. R) for offline analyses, while the CLAS software delivers auditory stimuli phase-locked to δ-wave peaks or troughs.

In the operating room (OR), the BIS index (Connor, 2022; Rampil, 1998), which monitors intraoperative hypnotic depth using 4 prefrontal electrodes, will be connected to the patient and anesthesia monitor. A 128-channel high-density EEG saline net (MagstimEGI) referenced to Cz will then be placed on the patient’s head (Figure 2). Hd-EEG impedances will be verified and maintained <50 kΩ for the study’s duration. The patient will be positioned supine, and all routine monitors will be installed, as per standard of care. The PMD200™ monitor, which assesses intraoperative pain levels through the NOL index using a finger sensor (Ben-Israel et al., 2013; Edry et al., 2016), will also be installed.

### 2.2 General protocol

#### 2.2.1 Anesthesia induction

**Figure 3:**
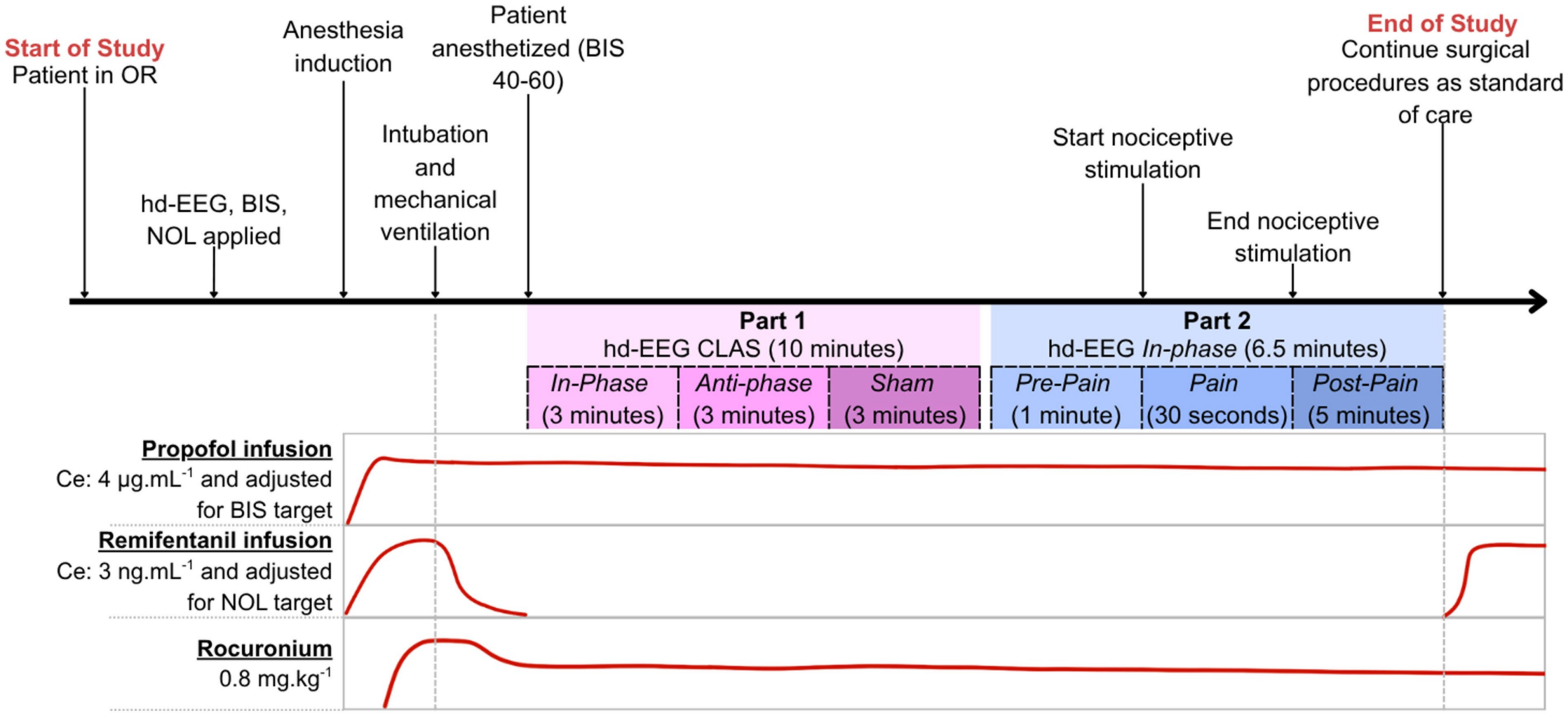
*Timeline of the study protocol*. Following baseline EEG recording in the awake state (5 min), general anesthesia is induced with propofol and remifentanil. Objective 1 includes three stimulation conditions (*in-phase, anti-phase, sham*; 3 min each, with 20 s pauses). Objective 2 involves *in-phase* stimulation with standardized nociceptive input (*Pre-Pain, Pain*, *Post-Pain* epochs). Anesthetic doses, BIS (target 40–60), and NOL (target <10) are maintained stable throughout.

The hd-EEG net will be connected to the amplifier. GA will be induced with propofol and remifentanil in target-controlled infusion (TCI) mode. Propofol target concentration at effect-site (Ce) will be set at 4 μg.mL^−1^ using the Schnider (Rampil, 1998) or Marsh (Marsh et al., 1991) model and adjusted to keep a BIS index value between 40 and 60. Remifentanil Ce will be set at 3 ng.mL^−1^ using the Minto model (Minto et al., 1997) to achieve a NOL index <10. To mitigate potential electromyographical interference with BIS and hd-EEG, rocuronium will be administered as a bolus dose of 0.8 mg.kg^−1^ once the patient no longer responds (Figure 3). Subsequently, endotracheal intubation will be performed, followed by initiation of mechanical ventilation. Remifentanil administration will be immediately halted after intubation upon cessation of painful stimuli. Impedance levels will be reassessed and adjusted to ensure they remain below 50 kΩ.

#### 2.2.2 Procedures for SA1 and SA2 (part 1)

Once anesthesia dosage is stable, BIS values are 40–60, predicted remifentanil Ce is <0.5 ng.mL-1 and the NOL is below 10, in-ear earphones (ER2XR Earphones; Etymotic Research, IL, United States) will be placed in patients’ ears, through which the CLAS auditory stimulation will be provided binaurally. These earphones already provide a 32–45 dB attenuation. To further attenuate ambient sounds in the OR, noise-blocking earmuffs (Peltor Optime 105; 3 M, MN, United States), effective up to 105 dB of ambient noise, will be placed over the earphones. The hd-EEG-CLAS software package will then deliver *in-phase*, *anti-phase*, and Rest (*sham*) stimulation blocks for 3 min each, with 20-s pauses between each stimulation type (10 min total). This interval is considered sufficient to prevent any carryover effects, as CLAS has shown only stimulus-locked effects, and do not persist beyond the targeted slow oscillatory cycles (Ngo et al., 2013; Ngo et al., 2015). Given that anesthetic doses will be stable and no CLAS carryover effect is expected beyond each stimulated wave, no randomization of stimulation blocks will be conducted. Under general anesthesia, cortical dynamics may gradually deepen over time despite stable TCI and by applying *in-phase* stimulation first, we adopt a conservative design that minimizes the risk of falsely attributing time-dependent anesthetic deepening to stimulation effects. Minimal physical contact will be made with the participant, and a minimal noise level will be maintained in the OR throughout the study for optimal isolation of the CLAS effects.

#### 2.2.3 Procedures for SA3 (part 2)

Immediately following the completion of Part 1, a 6.5 min *in-phase* stimulation block will be initiated, with simultaneous hd-EEG and pain monitoring using the NOL index, mean arterial pressure and heart rate. The first minute of *in-phase* stimulation will be free of painful stimulation (*Pre-Pain*). A standardized nociceptive stimulation will then be delivered for 30 s (*Pain*). This will consist in a standardized tetanic stimulation to the ulnar nerve of the non-dominant forearm delivered by a routine nerve stimulator (100 Hz, 70 mA; Life Tech - EZstim II) for a duration of 30 s. Measurements will continue during 5 min following painful stimulation (*Post-Pain*). Upon completion, noise-blocking earmuffs, in-ear earphones, and the hd-EEG net will be removed, and study team will exit the operating room. Surgery will begin and anesthetic procedures will continue as standard of care.

## 3 EEG data analysis

### 3.1 EEG pre-processing

EEG data will be preprocessed using a custom MNE Python script and will be bandpass filtered from 0.1 to 50 Hz. Non-scalp, noisy channels, and channels overlapping with the BIS monitor will be removed. EEG data will be re-referenced to an average reference. Data will then be segmented into the 6 conditions of study: (1) *In-phase*; (2) *Anti-phase*; (3) *sham*; (4) *Pre-Pain*; (5) *Pain*; (6) *Post-Pain*.

### 3.2 Separation of auditory-evoked responses from ongoing *δ*-wave activity

To dissociate auditory event-related potentials (ERPs) from modulation of ongoing δ-wave activity, we will adopt an approach previously validated in CLAS studies during sleep (Ngo et al., 2013). For each stimulation condition, EEG activity time-locked to auditory stimulus onset will be averaged, and the corresponding average from the *sham* condition will be subtracted to isolate stimulus-evoked responses. This procedure allows identification of early auditory ERP components while minimizing contamination from ongoing spontaneous slow oscillatory activity.

Importantly, primary analyses of δ-wave dynamics are based on individually detected δ-waves defined independently of stimulus-locked averaging. δ-wave detection and morphological metrics (amplitude, slope, transition frequency, density) are computed on continuous EEG signals using zero-crossing based criteria, ensuring that reported CLAS effects reflect modulation of endogenous oscillatory activity rather than purely evoked auditory responses.

### 3.3 δ-waves metrics

**Figure 4:**
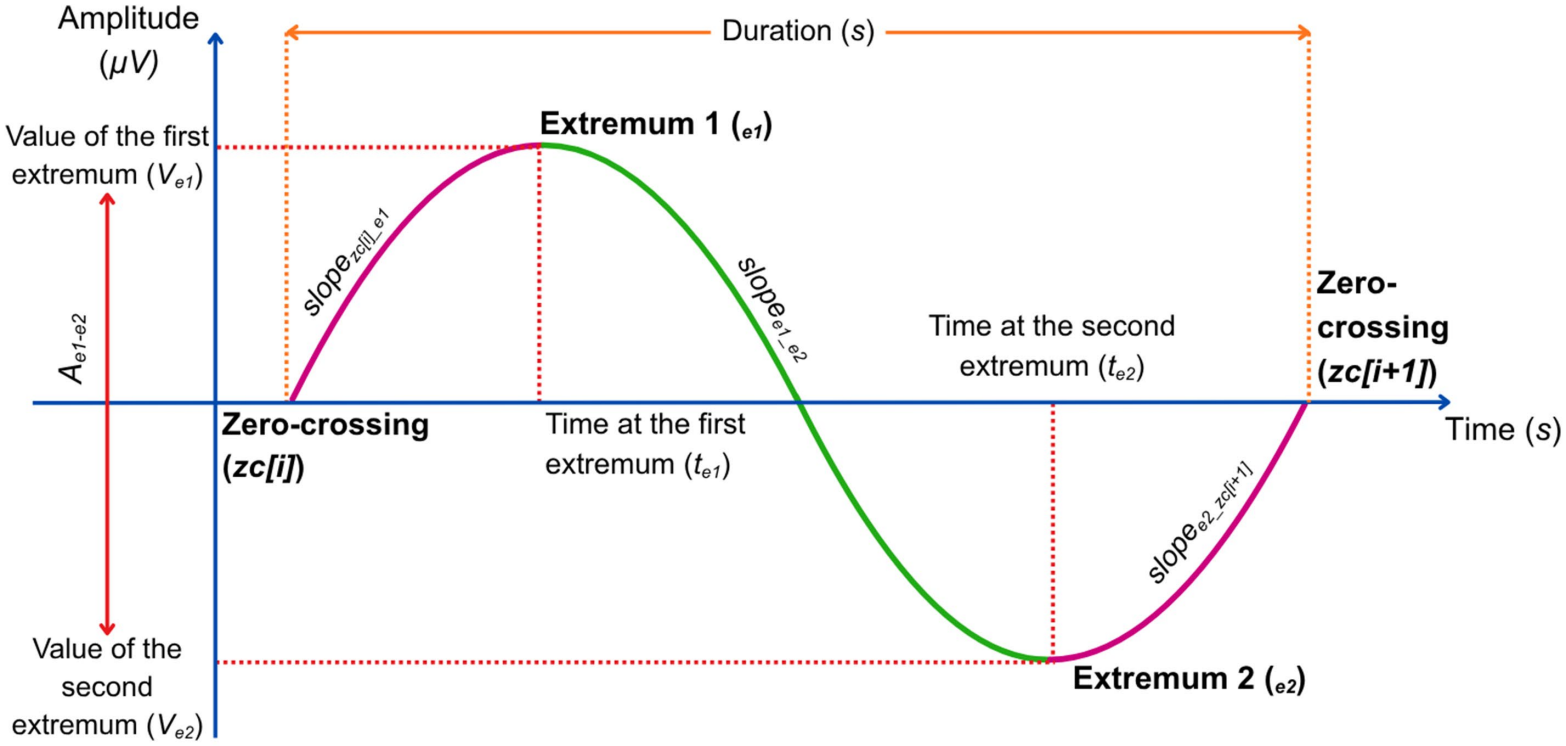
Delta-wave metrics. Quantification of δ-wave morphology. Each δ-wave is defined between three consecutive zero-crossings. Metrics include amplitude (difference between extremums, V_e1_–V_e2_), duration (time between first and last zero-crossings), slopes (rate of voltage change across segments), and transition frequency (inverse of twice the interval between extremums). These features provide sensitive markers of oscillatory dynamics under anesthesia and CLAS modulation.

Figure 4 illustrates some of the different metrics that are described in the following section. *Amplitude (μV):* The peak-to-peak amplitude was calculated as the absolute difference between the maximum and minimum values of the oscillation: $A_{e1-e2}=|V_{e1}-V_{e2}|$ , where *V_e1_* is the value of the first extremum in μV, and *V_e2_* is the value of the second extremum in μV. *Frequency (Hz)*: The mean frequency of the oscillation was defined as the inverse of its duration: $F=\frac{1}{d}$ . *Slopes (μV/s)*: We calculated three slope measures to quantify the rate of voltage change within each oscillation: (1) 1st zero-crossing to 1st extremum: $\text{slop}e_{\mathrm{zc}[i]\_e1}=\frac{|V_{e1}|}{t_{e1}}$ , (2) 1st extremum to 2nd extremum: $\text{slop}e_{e1\_e2}=\frac{A_{p-p}}{|t_{e1}-t_{e2}|}$ , (3) 2nd extremum to last zero-crossing: $\text{slop}e_{e2\_\mathrm{zc}[i+1]}=\frac{|V_{e2}|}{d-t_{e2}}$ , where *t_e1_* and *t_e2_* are the time (in seconds, relative to the initial zero-crossing) at which the first and second extremum occur, respectively. *Transition Frequency (Hz):* The transition frequency was defined as the inverse of twice the interval between the first extremum and the second extremum: $F_{\text{trans}}=\frac{1}{2|t_{e2}-t_{e1}|}$ . *Density (SOs/s)*: The density of delta waves (*δ*-waves) was defined as the number of δ-waves per second over the full duration of the recording: $\text{Density}=\frac{N_{\mathrm{\delta W}}}{T}$ , where *N_δW_* is the total amount of δ-waves detected in the recording, and *T* is the total duration of the recording in seconds.

### 3.4 Functional analysis

Beyond univariate analyses, we will characterize the large-scale neural dynamics underlying depth of anesthesia by constructing functional connectivity networks for each stimulation block. Connectivity will be computed using weighted phase lag index (wPLI) (Vinck et al., 2011), where EEG data will first be filtered into canonical frequency bands (delta 1–4 Hz, theta 4–8 Hz, alpha 8–14 Hz, beta 14–30 Hz).

The wPLI isdefined as: $\mathrm{wPL}I_{\mathrm{ij}}=\frac{E\{ℑ(C_{\mathrm{ij}})\}}{E\{|ℑ(C_{\mathrm{ij}})|\}}=\frac{E\{|ℑ(C_{\mathrm{ij}})|\mathrm{sgn}(ℑ(C_{\mathrm{ij}}))\}}{E\{|ℑ(C_{\mathrm{ij}})|\}}$ ,

where $ℑ(C_{\mathrm{ij}})$ is the imaginary part of cross-spectrum the imaginary component of the cross-spectrum between signals *i* and *j*. The cross-spectrum is defined as C_ij_ = Z_i_Z_j_, with Z_i_ and Z_j_ denoting the Fourier spectrum of channel *i* and the conjugate spectrum of channel *j*. The wPLI values range from 0 (no consistent lag/lead relationship) to 1 (perfect non-zero phase locking) (Vinck et al., 2011).

To assess the directionality of functional connectivity, we will compute the directed phase lag index (dPLI) (Stam and Van Straaten, 2012). The instantaneous phase of each EEG channel will be extracted using a Hilbert transform. For each time point *t*, the phase difference between channels *i* and *j* will be computed as $\Delta \phi_{t}=\phi_{i,t}-\phi_{j,t}$ .

The dPLI is then given by: $\mathrm{dPL}I_{\mathrm{ij}}=\langle H(\Delta_{\mathrm{\varphi t}})\rangle$ ,

where *H(x)* is the Heavyside step function: H(x)=1ifx>0,H(x)=0.5ifx=0,andH(x)=0 otherwise. dPLI values >0.5 indicate that channel *i* consistently leads channels *j*, whereas values <0.5 indicates the opposite.

### 3.5 Graph theoretical properties

For each frequency band, wPLI matrices will be thresholded into adjacency matrices, and graph-theoretical analyses will be performed. Core measures will include global efficiency (Latora and Marchiori, 2001), clustering coefficient (Watts and Strogatz, 1998), modularity (Newman, 2006), and small-worldness (Humphries and Gurney, 2008). Network hubs will be identified using node degree and betweenness centrality, reflecting the integration role of highly connected nodes (Van Den Heuvel and Sporns, 2013). Particular focus will be given to frontoparietal connectivity, which is strongly implicated in anesthetic-induced unconsciousness (Lee et al., 2013).

### 3.6 Micro-state analysis

To examine the fast temporal dynamics of large-scale cortical activity, we will perform an EEG microstate analysis. Microstates represent brief, quasi-stable scalp potential topographies thought to reflect coordinated activity within distributed neural networks (Lehmann et al., 1987; Michel and Koenig, 2018). Global field power (GFP) peaks will be identified from bandpass-filtered data, and the corresponding maps will be clustered using k-means to extract four canonical microstate classes (A-D) (Koenig et al., 2002). The continuous EEG will then be back-fitted to these templates to derive standard temporal parameters: mean duration, occurrence rate, coverage, and transition probabilities.

Comparisons across depth of anesthesia and stimulation blocks will reveal whether CLAS alters the stability or sequencing of cortical microstates, providing a complementary perspective to the spectral and connectivity analyses.

### 3.7 EEG and statistical analyses

#### 3.7.1 Effect of CLAS on *δ*-waves morphology and topography

EEG data will be processed at the level of individual channels to characterize the impact of CLAS on δ-wave morphology and topography using custom Python scripts. Each detected δ-wave will be segmented as a single event (250–10,000 ms duration), and morphology will be quantified by amplitude, slope, transition frequency, density, and power spectral density (Bouchard et al., 2021). These metrics will be compared across conditions (*in-phase*, *anti-phase, sham*) and cortical regions of interest (frontal, central, midline, parietal, temporal, occipital). Descriptive statistics will be computed at both the subject and group level, with comparisons assessed using repeated-measures ANOVA and independent-samples *t*-tests, including age and sex as covariates.

Topographical EEG maps will be generated for canonical frequency bands (delta (0.1–4 Hz), theta (4–8 Hz), alpha (8–13 Hz), beta (13–30 Hz)), and phase-amplitude coupling measures will be calculated to further assess condition-dependent differences in oscillatory dynamics. Topographical mapping will allow testing for regional differences, as frontal predominance of *δ*-waves is a hallmark of both sleep and propofol anesthesia (Murphy et al., 2011; Purdon et al., 2015). To extend beyond scalp measures and as an exploratory aim, source estimation will be performed using standardized head models (Michel and He, 2019). Finally, propagation analyses will be conducted to quantify directionality of δ-wave travel across the cortex, as slow oscillations are known to spread as traveling waves and have been proposed as indices of large-scale cortical communication (Massimini et al., 2004; Tononi and Massimini, 2008).

#### 3.7.2 Association of CLAS with depth of anesthesia

Depth of anesthesia will be evaluated using the BIS index, an EEG-derived index validated for intraoperative monitoring (Connor, 2022; Rampil, 1998). BIS index values will be compared across *in-phase*, *anti-phase*, and *sham* blocks using repeated-measures ANOVA and pairwise paired *t*-tests with Holm–Bonferroni correction, to control for multiple comparisons. To probe the relationship between the BIS index and underlying cortical dynamics, correlation analyses will be run between BIS values and *δ*-wave features (amplitude, slope, spectral power), building on prior evidence that BIS partly reflects the presence and strength of slow oscillatory activity (Akeju and Brown, 2017; Bruhn et al., 2000). To further dissociate oscillatory modulation from global state-dependent spectral changes, the aperiodic (1/f) parametrization will also be extracted for each block and examined in relation to BIS values (Donoghue et al., 2020; Lendner et al., 2020).

The impact of CLAS large-scale neural dynamics during general anesthesia will also be quantified through EEG-derived network measures, including functional connectivity (wPLI, Vinck et al., 2011; dPLI, Stam and Van Straaten, 2012) and graph-theoretical properties (network motifs; degree and direction of frontoparietal connectivity; Lee and Mashour, 2018). We will also investigate the effect of CLAS on EEG microstates in order to characterize the temporal dynamics of whole-brain neuronal network.

#### 3.7.3 CLAS and nociceptive stimulation

Because nociceptive stimulation will be delivered only during *in-phase*, the primary analyses will assess within-subject changes across the *Pre-Pain*, *Pain*, and *Post-Pain* epochs. As a first step, *δ*-wave morphology will be quantified by comparing amplitude, slope, and power spectral density (PSD) across the three conditions, since these features provide sensitive markers of cortical arousal and nociceptive disruption under anesthesia (Bonhomme et al., 2016; Plourde et al., 2006). To dissociate band-limited power changes from broadband spectral shifts that can occur under propofol anesthesia, we will additionally parameterize the aperiodic (1/f-like) component of the EEG power spectrum for each stimulation block. Power spectral density will be estimated per block and channel, and the aperiodic exponent (slope) and offset will be extracted over a predefined frequency range using spectral parameterization methods. These aperiodic parameters will be compared across *in-phase, anti-phase*, and *sham* conditions and, where relevant, included as covariates when interpreting *δ*-band power changes.

The primary analysis of nociceptive reactivity will be changes in the NOL index and time-to-recovery. Secondary analysis will examine changes in mean arterial pressure (MAP), heart rate (HR), and BIS index values. Source localization and propagation analyses will further characterize the cortical generators and spatiotemporal spread of δ-waves during nociception (Massimini et al., 2004; Murphy et al., 2011).

Statistical models will include linear mixed-effects analyses with Condition (*Pre-Pain*, *Pain*, *Post-Pain*) as a fixed factor and age, and sex. *p*-values will be corrected for multiple comparisons using familywise error correction. This design enables testing whether *in-phase* CLAS can preserve *δ*-wave integrity and stabilize autonomic reactivity in the presence of nociceptive input, a key criterion for balanced anesthesia (Garcia-Larrea and Bastuji, 2018).

## 4 Anticipated results

As this is the first protocol to apply CLAS during general anesthesia, the following section outlines hypothesis-driven expectations grounded in existing literature on sleep and functional connectivity, rather than empirically informed predictions.

### 4.1 Primary hypothesis (H1): phase-specific modulation of SWA by CLAS

We anticipate phase-specific effects of CLAS on *δ*-wave dynamics during propofol anesthesia. *In-phase* CLAS should enhance δ-wave morphology and power relative to *sham*, increasing peak-to-peak amplitude, steepening slopes between extrema, prolonging, and augmenting PSD in 0.1–4 Hz, consistent with sleep (Ngo et al., 2013; Ngo et al., 2015; Zeller et al., 2024). *Anti-phase* CLAS is expected to disrupt *δ*-wave expression (reduced amplitude, flatter slopes, shorter duration, lower PSD), reflecting the directional nature of phase-locking (Esfahani et al., 2023). Topographically, frontal predominance is expected for *in-phase* CLAS effects (Murphy et al., 2011; Purdon et al., 2015), and source/propagation analyses should implicate prefrontal-cingulate generators and anterior to posterior traveling waves typical of unconscious states (Massimini et al., 2004).

### 4.2 Secondary hypothesis (H2): CLAS and depth of anesthesia

We expect CLAS to modulate depth of anesthesia as measured by the BIS. BIS index values are predicted to remain within the safe surgical range (40–60) across all conditions (Sessler et al., 2012; Lewis et al., 2019), but *in-phase* CLAS may be associated with slightly lower BIS values, reflecting enhanced slow-wave synchronization, while *anti-phase* CLAS may transiently elevate BIS values by disrupting oscillatory regularity. Correlation analyses are expected to show that BIS fluctuations covary with *δ*-wave amplitude and slope, consistent with evidence that BIS partly reflects slow oscillatory strength (Akeju and Brown, 2017; Bruhn et al., 2000). BIS will be interpreted as an EEG-derived clinical correlate, and not a direct consciousness measure.

### 4.3 Secondary hypothesis (H3): CLAS effects on SWA under nociception

Under nociceptive input, *in-phase* CLAS is expected to partially enhance *δ*-wave dynamics relative to pain without CLAS, though the magnitude of enhancement will be attenuated compared to non-nociceptive conditions. Specifically, under *in-phase* CLAS, δ-wave morphology should recover faster in *Post-Pain*, with amplitude/PSD returning closer to *Pre-Pain* values than expected absent stimulation. At the network level, nociception may interrupt anterior to posterior spread and temporarily reduce inter-regional coherence, whereas *in-phase* CLAS should stabilize these spatiotemporal dynamics, limiting disruption of fronto-central coherence. We also hypothesize that *in-phase* CLAS will buffer autonomic nociceptive responses, yielding smaller increases in NOL, MAP, and HR during tetanic stimulation and faster return toward baseline afterward (Ben-Israel et al., 2013; Edry et al., 2016), without altering anesthetic/analgesic dosing.

### 4.4 Network-level and microstate predictions

Functional connectivity analyses are expected to reveal more locally coherent *δ*-band activity but reduced large-scale integration during *in-phase* CLAS. Specifically, we anticipate decreased frontoparietal connectivity and lower global efficiency, accompanied by increased modular segregation and modest reductions in small-worldness relative to *anti-phase* and *sham* conditions. This pattern would extend prior findings that anesthetic-induced unconsciousness is associated with breakdown of large-scale network integration, particularly within fronto-parietal circuits (Boveroux et al., 2010; Bonhomme et al., 2016; Lee and Mashour, 2018), and would test whether strengthening δ-waves further accentuates this network fragmentation.

At the level of EEG microstates, we expect *in-phase* CLAS to be associated with longer mean microstate durations, increased coverage of “unconsciousness-associated” classes, and a reduced repertoire of transitions compared with *anti-phase* and *sham* stimulation (Koenig et al., 2002; Lehmann et al., 1987; Michel and Koenig, 2018). Such changes would indicate greater temporal stability of large-scale cortical states, complementing spectral and connectivity markers of a more deeply unconscious network configuration.

## 5 Discussion

The present study proposes a novel integration of closed-loop auditory stimulation (CLAS) into the field of anesthesiology. While balanced anesthesia has historically relied on pharmacological synergies (Lundy, 1926; Sessler et al., 2012), our design explores whether a non-pharmacological intervention can contribute to this balance by directly modulating *δ*-wave activity under propofol anesthesia. If confirmed, these results would position CLAS as the first neuromodulatory techniques capable of directly amplifying anesthetic δ-waves, with the potential to stabilize EEG signatures associated with anesthetic brain states without increasing anesthetic dosage. This addresses longstanding concerns about cardiovascular instability, delayed recovery, and burst suppression at high drug concentrations (Purdon et al., 2013; Willingham et al., 2014).

CLAS has been shown to enhance slow oscillations (SOs) and slow-wave activity (SWA) during natural sleep (Ngo et al., 2013; Malkani and Zee, 2020; Zeller et al., 2024) and more recently during pharmacological sedation (Smith et al., 2024). However, its efficacy during propofol GA, which is a deeper, pharmacologically stabilized state that routinely includes nociceptive input, remains unexplored. Evidence from EEG and intracranial recordings demonstrates that auditory processing persists even during deep anesthesia (Plourde et al., 2006), providing a mechanistic foundation for CLAS interact with ongoing cortical dynamics. If peak-synchronized stimulation enhances δ-wave amplitude and trough-synchronized stimulation disrupts it, as shown in sleep studies (Esfahani et al., 2023; Ngo et al., 2013), CLAS could become the first non-pharmacological method for directly amplifying anesthetic *δ*-waves during general anesthesia, with implications for reducing hypnotic drug requirements.

An essential translational step is testing whether CLAS retains its neuromodulatory capabilities under nociceptive stimulation. Nociceptive input activates ascending thalamocortical pathways and can transiently fragment *δ*-waves, thereby destabilizing hypnosis (Bonhomme et al., 2016; Plourde et al., 2006). By incorporating standardized pain during peak-synchronized CLAS, the present study evaluates whether oscillatory reinforcement persists in conditions that approximate surgical anesthesia. Confirmation of this effect would demonstrate that CLAS can buffer against nociception-induced cortical arousals and support balanced anesthesia by stabilizing both hypnotic depth (BIS) and nociceptive reactivity (NOL).

Another distinctive feature of this work is the integration of closed-loop stimulation with high-density EEG. The dense spatial sampling allows for detailed characterization of δ-wave morphology, cortical propagation, and source localization (Massimini et al., 2004). Moreover, functional connectivity and graph-theoretical analyses provide a systems-level perspective on anesthetic brain states. Prior studies show that anesthesia disrupts frontoparietal communication, reduces small-world topology, and fragments large-scale networks (Boveroux et al., 2010; Lee and Mashour, 2018). By contrast, peak-synchronized CLAS may increase global efficiency and preserve small-world properties (Latora and Marchiori, 2001; Watts and Strogatz, 1998), supporting the hypothesis that reinforced δ-waves enhance large-scale cortical coordination. This approach therefore not only evaluates the anesthetic utility of CLAS but also tests theoretical models of consciousness by probing the causal role of slow oscillations in sustaining unconsciousness (Akeju and Brown, 2017; Tononi and Massimini, 2008).

If our central hypothesis is confirmed, the implications are twofold. Clinically, CLAS could provide anesthesiologists with a neuromodulatory tool to deepen unconsciousness and stabilize responses to nociception without increasing drug dosage, an approach particularly relevant for vulnerable populations such as elderly patients or those with hemodynamic fragility. Scientifically, anesthesia offers a reversible, controlled model of unconsciousness, making it an ideal context for testing whether δ-waves are mechanistic contributors rather than passive markers of loss of consciousness. Whether CLAS-induced amplification of δ-waves correlates with BIS stabilization, preserved network integration, and reduced nociceptive responses will directly inform this debate.

## 6 Limitations

Several limitations should be acknowledged. First, the study design is restricted to neurologically healthy surgical patients, which may limit generalizability to vulnerable populations, where anesthetic management is most challenging. Second, the inclusion of a broad age range (18–80 years) was necessary to facilitate recruitment in a clinical intraoperative setting but may introduce age-related neurophysiological and pharmacokinetic variability. To mitigate this, the study employs a within-subject design and includes age as a covariate in statistical analyses; if sampling permits, this cohort may also enable exploratory analyses of age-related differences in CLAS responsiveness under anesthesia. Third, although high-density EEG offers improved spatial resolution compared to standard EEG, source localization remains constrained by the absence of individual MRI head models; therefore, source localization and propagation findings should be interpreted cautiously (Custo et al., 2017). This reflects a feasibility-driven decision, as requiring an additional MRI visit in a surgical population would substantially increase participant burden and limit recruitment; future studies may incorporate individual MRI to enable more precise localization. Fourth, auditory stimuli may be variably attenuated by earphones and earmuffs, and inter-individual differences in auditory sensitivity under anesthesia could influence responsiveness to CLAS. Finally, the protocol focuses on short stimulation blocks and acute nociceptive challenges; the long-term impact of CLAS on anesthetic requirements, recovery profiles, or postoperative outcomes remains untested.

## 7 Future directions

Future research should extend this paradigm in several directions. Larger trials will be required to confirm whether CLAS can reduce propofol or opioid doses without compromising anesthetic stability. Parallel studies in vulnerable populations (elderly, septic, or hemorrhagic patients) could evaluate its potential to mitigate risks during these specific surgeries. Beyond anesthesia, CLAS could serve as a broader neuromodulatory tool to probe the causal role of δ-waves in consciousness, with applications to disorders of consciousness, sleep disorders, and neurodegenerative conditions where SWA is impaired (Baglioni et al., 2016; Scullin, 2013). Ultimately, demonstrating that non-pharmacological reinforcement of δ-waves can safely stabilize unconsciousness and nociceptive balance would establish CLAS as a transformative method in both clinical anesthesiology and systems neuroscience.
